# Immunoinflammatory Mechanisms Connecting Periodontitis and Solid Tumors: A Systematic Review of Original Evidence

**DOI:** 10.7759/cureus.96491

**Published:** 2025-11-10

**Authors:** Houda El Ayachi, Imad Barjij, Amine Cherkaoui

**Affiliations:** 1 Department of Dental Sciences, Faculty of Medicine, Pharmacy and Dental Medicine, Fez Sidi Mohamed Ben Abdellah University, Fez, MAR; 2 Department of Medical Oncology, National Institute of Oncology, Ibn Sina University Hospital, Rabat, MAR; 3 Department of Medical Oncology, Faculty of Medicine and Pharmacy, Mohammed V University, Rabat, MAR; 4 Department of Periodontology, Faculty of Dental Medicine, Mohammed V University, Rabat, MAR

**Keywords:** carcinogenesis, immune response, immunoinflammatory mechanisms, inflammation, microbiota, periodontitis, solid tumors, systemic health

## Abstract

Mounting evidence indicates that periodontitis may play a contributory role in the pathogenesis of systemic diseases, including cancer, primarily through mechanisms involving chronic inflammation and immune dysregulation. Nonetheless, the specific immunoinflammatory pathways that underpin the relationship between periodontitis and solid tumors remain only partially elucidated. To conduct a systematic review of original clinical studies that examine the immunoinflammatory mechanisms potentially linking periodontitis with the initiation or progression of solid malignancies.

Adhering to the Preferred Reporting Items for Systematic Reviews and Meta-Analyses (PRISMA) 2020 framework, we conducted a comprehensive literature search across four major databases (PubMed, Scopus, Web of Science, and Embase) through March 2025. Eligible studies were limited to original human research that investigated the association between periodontitis and solid tumors with an emphasis on immunological or inflammatory biomarkers. Following a rigorous screening process, 19 studies met the inclusion criteria and were analyzed.

A consistent body of evidence across the included studies suggests a significant association between periodontitis and heightened risk or severity of various solid tumors, notably colorectal, pancreatic, lung, prostate, and breast cancers. Common immunoinflammatory signatures observed among affected individuals included elevated systemic levels of cytokines, such as IL-6 and TNF-α, altered immune cell profiles characterized by increased regulatory T-cells, and evidence of microbial translocation involving pathogens, including *Porphyromonas gingivalis* and *Fusobacterium nucleatum*. Despite variability in study methodologies, the overall quality of evidence was predominantly rated as moderate. These findings support the hypothesis that chronic periodontal inflammation may act as a systemic immunoinflammatory driver of carcinogenesis.

Periodontitis is increasingly recognized as a potential contributor to solid tumor development via converging immunoinflammatory mechanisms. These include sustained systemic inflammation, microbial dysbiosis, and immune modulation. Although causal inferences cannot yet be made, the biological plausibility and coherence across studies underscore the necessity for more detailed mechanistic and longitudinal investigations. Integration of periodontal evaluation into broader healthcare strategies may open novel avenues for cancer prevention and systemic disease management.

## Introduction and background

Chronic inflammation is increasingly recognized as a pivotal contributor to the initiation and progression of various solid tumors. It orchestrates several oncogenic processes, including DNA damage, uncontrolled cellular proliferation, angiogenesis, and immune evasion. Among the systemic inflammatory conditions implicated in this context, periodontitis has garnered growing attention due to its capacity to influence distant organs through both immunological and microbial mechanisms [1-4].

Periodontitis is a widespread inflammatory disease targeting the tooth-supporting structures. Its pathogenesis involves a dysregulated host immune response to a disrupted oral microbiome. Beyond the local destruction of periodontal tissues, the condition induces a state of persistent, low-grade systemic inflammation, characterized by the release of proinflammatory cytokines, the translocation of microbial products into the bloodstream, and sustained alterations in immune regulation [1,5-7].

Accumulating observational data suggest a potential association between periodontitis and an elevated risk of various solid tumors, including those of the colon, lung, pancreas, breast, and upper aerodigestive tract. While the magnitude of these associations is generally moderate, their biological plausibility is supported by shared pathogenic pathways such as chronic inflammation, compromised immune surveillance, and microbial dissemination [8-11].

Central to this interplay are the immunoinflammatory cascades initiated in the periodontal niche and perpetuated systemically. Periodontitis is associated with persistent elevations of circulating cytokines, notably interleukin-6 (IL-6), tumor necrosis factor-alpha (TNF-α), and C-reactive protein (CRP), which are factors known to facilitate tumorigenesis through enhanced proliferation, resistance to apoptosis, and increased metastatic potential. Concurrent immune dysregulation is also evident, including skewed immune cell profiles, increased regulatory T-cell activity, and impaired innate responses, which together contribute to a pro-tumorigenic systemic environment [1,3,6,7].

Moreover, certain periodontopathogens such as *Porphyromonas gingivalis* and *Fusobacterium nucleatum* have been implicated in tumorigenic processes. These organisms may interfere with host cell signaling, inhibit apoptosis, or promote immune environments favorable to neoplastic transformation. Additionally, microbial byproducts originating from the periodontal environment may disseminate systemically, disrupting epithelial integrity and immune equilibrium at distant sites [4,12-14].

The insidious and chronic nature of periodontitis allows these immunoinflammatory effects to persist over time, potentially priming the host for oncogenic changes. The notable convergence of biological pathways involved in both periodontal disease and tumor development suggests a systematic biological interconnection, warranting deeper investigation [1,3,4,13].

Despite growing interest in the oral-systemic health axis, most prior reviews have focused on epidemiological correlations or emphasized microbial associations. Less attention has been given to the synthesis of original evidence describing the immunological and inflammatory mechanisms that may bridge periodontitis and cancer [4,8,13,15].

This systematic review aims to address that gap by consolidating primary clinical data exploring these mechanisms. It provides a targeted analysis of relevant studies, emphasizing the consistency and strength of the evidence, the biological pathways implicated, and the broader implications for understanding cancer as a process that oral inflammatory conditions may influence.

## Review

Methodology

Protocol and Registration

This systematic review was conducted in accordance with the Preferred Reporting Items for Systematic Reviews and Meta-Analyses (PRISMA) 2020 guidelines. The review protocol was developed prospectively and provided a structured framework for each stage of the review, including the formulation of the search strategy, definition of selection criteria, procedures for data extraction, and synthesis of findings. No formal registration was submitted to PROSPERO or any other registry.

Eligibility Criteria

To be included in this systematic review, studies were required to meet several predefined criteria. Only original research involving human participants was considered eligible for inclusion. Specifically, studies had to adopt an observational design, including cohort, case-control, or cross-sectional methodologies. Each study needed to investigate periodontitis as the primary exposure variable and report at least one solid tumor as the clinical outcome. Crucially, the studies also had to include an explicit evaluation or discussion of immunoinflammatory mechanisms as potential biological links between periodontitis and cancer development.

Studies that did not meet these conditions were excluded. This applied to review articles, animal studies, editorials, and conference abstracts. Furthermore, to ensure methodological rigor, only studies published in peer-reviewed journals were included in the final analysis.

Information Sources

A comprehensive search was conducted across multiple electronic databases, including PubMed (via MEDLINE), Scopus, Web of Science, and Embase. The final literature search was completed in March 2025. Reference lists of included studies were manually screened to identify additional relevant publications.

Search Strategy

The search strategy integrated free-text terms and controlled vocabulary (e.g., MeSH terms) relevant to periodontitis, solid tumors, and immunoinflammatory pathways. Search terms included combinations such as “periodontal disease,” “cancer,” “solid tumors,” “inflammation,” “immune response,” “cytokines,” and “mechanisms.” Boolean operators (AND/OR) were employed to enhance the comprehensiveness and sensitivity of the search.

Study Selection

Two reviewers independently screened all retrieved records at the title and abstract levels. Full texts of potentially eligible articles were then assessed for inclusion. Any discrepancies were resolved through discussion and consensus.

Out of 1243 identified records, 203 duplicates were removed, and 1040 unique titles and abstracts were screened. After a full-text review of 67 articles, 19 studies met the inclusion criteria and were selected for retention. The selection process is illustrated in the PRISMA flow diagram.

Data Collection Process

For each included study, relevant data were extracted into a standardized spreadsheet by one reviewer and subsequently verified by a second. The data extraction framework captured information such as author, publication year, country, study design, sample size, cancer type, periodontal assessment method, inflammatory markers examined, key findings, proposed biological mechanisms, and overall level of evidence.

Data Items

Data elements were defined a priori in alignment with the review objectives. Special attention was given to the method of periodontal diagnosis (clinical or radiographic), the types of solid tumors investigated, and the specific immune or inflammatory biomarkers assessed (e.g., IL-6, regulatory T-cells, bacterial antigens).

Risk of Bias and Evidence Level

Risk of bias was formally assessed using validated tools adapted to the study design. The Newcastle-Ottawa Scale (NOS) [16] was applied to all cohort and case-control studies, while the AXIS tool [17] was used for cross-sectional studies. Each included study was independently reviewed by two authors, and discrepancies were resolved by consensus. Risk levels were classified as low, moderate, or high. This structured approach strengthens the transparency and reproducibility of our synthesis, addressing prior limitations in narrative-only bias assessments.

Synthesis of Results

Given the heterogeneity of study designs, populations, and outcome metrics, a meta-analysis was not performed. Instead, findings were synthesized narratively, organized by immune marker type or mechanistic theme, and compared across different types of cancer. Results are presented in structured tables and figures, highlighting key mechanistic links and hypothesized biological pathways.

Results

Overview of Included Studies

**Figure 1:**
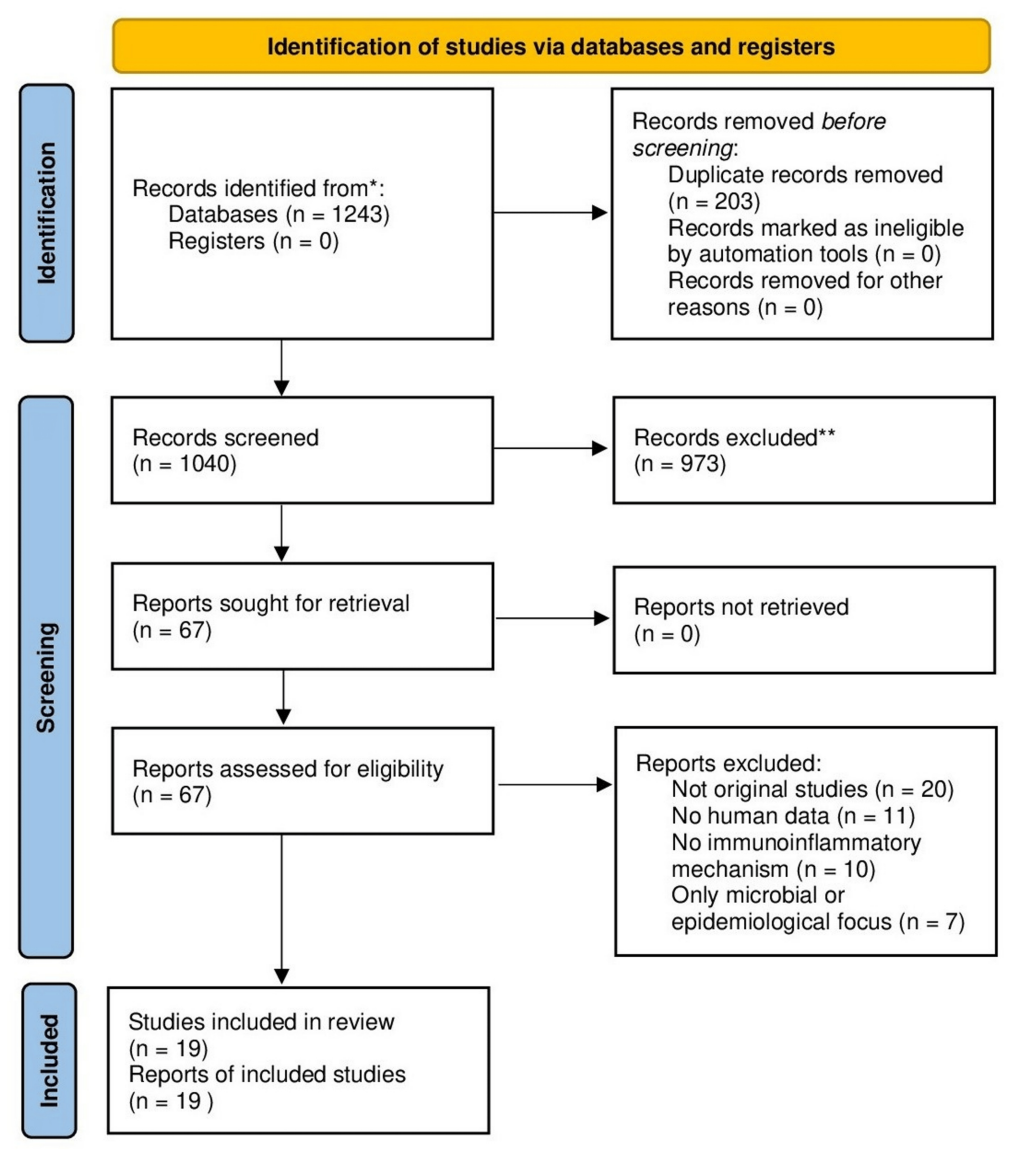
PRISMA flow diagram indicating the selection process of the articles in the present systematic review The diagram illustrates the number of records identified, screened, excluded, and finally included in the qualitative synthesis. PRISMA: Preferred Reporting Items for Systematic reviews and Meta-Analyses

A total of 1243 records were identified through four databases. After removing duplicates and screening titles/abstracts, 67 full-text articles were evaluated, and 19 studies were included. The flow of study selection is presented in Figure 1.

Nineteen original studies met all inclusion criteria following full-text review. These studies represented a diverse geographical and methodological spectrum, encompassing various populations and solid tumor types. Most included studies were either prospective or retrospective cohort designs, supplemented by a smaller number of case-control and cross-sectional studies. Sample sizes varied widely, ranging from fewer than 100 participants to national cohorts comprising more than 700,000 individuals.

The relationship between periodontitis and a range of solid malignancies was explored, including colorectal, pancreatic, lung, breast, prostate, esophageal, and oropharyngeal cancers. Some studies adopted a broad approach by examining associations with “all solid tumors.” In contrast, others focused on specific anatomical sites or employed surrogate markers for cancer outcomes, such as prostate-specific antigen (PSA) levels or mortality data.

Diagnostic approaches for periodontitis were heterogeneous and included clinical assessments (e.g., probing depth, clinical attachment loss), radiographic evaluations, and self-reported diagnoses. In certain studies, additional diagnostic methods involved microbiological analysis or measurement of immune responses, such as antibody titers against periodontal pathogens.

A detailed overview of the 19 included studies, including study design, sample size, diagnostic methods, cancer type, immune markers, and principal outcomes, is provided in Table 1.

**Table 1:** Data extracted from all the studies included in the present systematic review PD: periodontal disease, CDC: Centers for Disease Control and Prevention, AAP: American Academy of Periodontology, EFP: European Federation of Periodontology, CAL: clinical attachment level, PI: plaque index, GI: gingival index, PSA: prostate-specific antigen, CRP: C-reactive protein, IL: interleukin, TNF-α: tumor necrosis factor alpha, TLR: toll-like receptor, ESCC: esophageal squamous cell carcinoma, IHC: immunohistochemistry, qPCR: quantitative polymerase chain reaction, NHANES: National Health and Nutrition Examination Survey, ARIC: atherosclerosis risk in communities study, HPFS: health professionals follow-up study, Treg: regulatory T-cell, Kgp: lysine-specific gingipain (*Porphyromonas gingivalis*), *F. nucleatum: Fusobacterium nucleatum, A. actinomycetemcomitans: Aggregatibacter actinomycetemcomitans, *CRC: colorectal cancer, HR: hazard ratio, OR: odds ratio, RR: relative risk

Author (year)	Country	Study design	Sample size	Cancer type	Periodontal diagnosis	Inflammatory/immune markers	Main findings	Suggested causal link	Evidence level
Ahn et al. (2012) [12]	USA	Prospective cohort (NHANES III)	12,605 (periodontal exam); 7,852 (*P. gingivalis* IgG)	Orodigestive cancers (colorectal, pancreatic)	CDC/AAP criteria; serum *P. gingivalis* IgG	Serum *P. gingivalis* IgG antibody	Periodontitis associated with increased orodigestive cancer mortality (RR=2.28); *P. gingivalis* exposure linked to mortality even without clinical PD (RR=2.25)	Systemic inflammation from periodontal pathogens; microbial-driven carcinogenesis	Moderate
Kajihara et al. (2022) [3]	Japan	Cross-sectional observational clinical study	61 patients with PD and cancer	Cancer (non-specific) with focus on immune suppression markers	Clinical diagnosis + cytokine and Treg analysis	Treg cells, IL-6 via flow cytometry	Higher Treg/IL-6 levels in the PD+ cancer group; Tregs enriched in patients with history of both periodontitis and cancer	Immune evasion and systemic inflammation via Treg activation in the tumor microenvironment	Moderate
Kim et al. (2022) [18]	South Korea	Retrospective cohort (national health database)	713,203 adults	All solid tumors	CPI-based classification	Not measured directly	Increased cancer risk in PD patients across multiple organ systems	Chronic inflammation and systemic immune activation	Moderate
Mai et al. (2016) [19]	USA	Prospective cohort (Buffalo OsteoPerio)	1,337 postmenopausal women	Lung cancer	Radiographic bone loss (alveolar)	Not measured	Severe periodontitis associated with increased lung cancer risk	Microbial dysbiosis and low-grade inflammation influencing systemic oncogenesis	Moderate
Ortiz et al. (2022) [20]	USA (Puerto Rico)	Cross-sectional clinical study	134 Hispanic adults	Oral/oropharyngeal cancer (indirect via taxa)	CDC/AAP criteria	Salivary 16S rRNA sequencing	Enrichment of cancer-associated taxa (e.g., Prevotella, Treponema) in patients with severe periodontitis	Microbiota-driven inflammation linked to cancer-prone dysbiosis	Moderate
Qi et al. (2020) [21]	USA	Longitudinal cohort (NHANES III, 23 years)	6,491 cancer- and CVD-free at baseline	None measured directly (mortality proxy)	IgG antibodies to 19 periodontal pathogens	IgG antibody cluster profiles	Specific pathogen clusters associated with all-cause mortality; overlap with cancer-linked pathogens	Immune exposure to PD pathogens associated with systemic disease and mortality	Moderate
Saleh et al. (2025) [22]	USA	Retrospective cross-sectional (NHANES 2001–2010)	3,020 men (≥30 years)	Prostate cancer (PSA as surrogate)	EFP/AAP + CDC/AAP; pocket depth + tooth loss	PSA, CRP	Marginal correlation between PD stage × PD depth and PSA; edentulism associated with future prostate treatment	Inflammation from PD may impact the prostate tissue environment	Moderate
Uçan Yarkaç et al. (2024) [23]	Turkey	Cross-sectional clinical study	120 (60 young, 60 elderly)	None (focus on aging + inflammation)	Clinical indices: PD, CAL, PI, GI	IL-17, IL-18, TNF-α, TLR2, TLR4 (saliva via ELISA)	Inflammatory cytokines and TLRs increased with age and PD severity	Age amplifies the inflammatory host response in PD	Moderate
Heikkilä et al. (2018) [24]	Finland	Register-based cohort (10-year follow-up)	68,273 adults	All cancers, focus on the pancreas	Inferred from treatment codes	None directly measured	PD linked to increased cancer mortality; strongest for pancreatic cancer (RR=2.32)	Chronic inflammation may raise systemic cancer susceptibility	Moderate
Gao et al. (2016) [25]	China	Case-control study (IHC and qPCR in tumor tissues)	100 ESCC patients, 30 controls	ESCC	Presence of *P. gingivalis* in tissue samples	Kgp protease, 16S rRNA, IHC expression	*P. gingivalis* detected in 61% of tumor tissues vs. 0% of controls; associated with poor prognosis	Microbial-driven immune suppression in the local tumor niche	Moderate
Fan et al. (2018) [26]	USA	Nested case–control in a prospective cohort	361 pancreatic cancer cases, 371 controls	Pancreatic cancer	Microbial oral profile (16S rRNA; no clinical PD diagnosis)	*P. gingivalis*, *A. actinomycetemcomitans*, *Fusobacteria*	*P. gingivalis* (OR=1.60) and *A. actinomycetemcomitans* (OR=2.20) associated with higher pancreatic cancer risk; *Fusobacteria* protective (OR<1)	Oral microbial dysbiosis contributes to pancreatic carcinogenesis via immune modulation	Moderate
Chang et al. (2016) [27]	Taiwan	Nationwide retrospective cohort	214,890 subjects	Pancreatic cancer	ICD-9 diagnosis of periodontitis	None measured directly	PD patients had higher risk of pancreatic cancer (HR=1.55); association stronger among elderly (HR=2.17 for ≥65 years)	Chronic periodontitis linked to systemic cancer via inflammatory pathways	Moderate
Chung et al. (2016) [9]	Taiwan	Nationwide retrospective matched cohort	80,280 (40,140 PD, 40,140 controls)	All solid cancers (especially GI, genitourinary, oral)	Chronic periodontitis by ICD codes	None measured directly	PD associated with increased risk of overall cancer (HR=1.23); strongest associations for genitourinary (HR=1.30) and GI cancers (HR=1.23)	Systemic immune impact of chronic oral inflammation	Moderate
Castellarin et al. (2012) [28]	Canada	Case-control metagenomic tumor study	99 colorectal tumors	Colorectal cancer	*F. nucleatum* presence (no clinical PD evaluation)	RNA-seq and qPCR of tumor vs normal mucosa	*F. nucleatum* highly enriched in tumor tissues (mean 79×); associated with lymph node metastasis	Oral anaerobes translocate and modulate the tumor microenvironment	Moderate
Freudenheim et al. (2016) [29]	USA	Prospective cohort (Women’s Health Initiative)	73,737 postmenopausal women	Breast cancer	Self-reported periodontal disease	None measured	14% increased risk of invasive breast cancer (HR=1.14); strongest effect among recent ex-smokers (HR=1.36)	Chronic PD and tobacco act synergistically to enhance breast cancer risk	Moderate
Hu et al. (2018) [30]	Taiwan	Nationwide retrospective cohort	106,487 PD patients, 106,487 controls	Colorectal cancer	ICD codes (523.0–523.4); visits/year used as severity proxy	None measured directly	PD associated with increased CRC risk (HR=1.64); risk higher with >10 dental visits/year (HR=1.78)	Repeated PD activity may drive systemic dysbiosis and colonic inflammation	Moderate
Michaud et al. (2018) [31]	USA	Prospective cohort (ARIC study)	7,466 subjects, 1,648 cancers	Lung, colorectal, total (not breast/prostate)	Clinical exam (CAL, PD); CDC-AAP criteria	None measured	Severe PD increased total cancer risk (HR=1.24); strong association with lung (HR=2.33) and colorectal (HR=1.51)	Oral inflammation drives systemic cancer-prone environments	Moderate
Momen-Heravi et al. (2017) [32]	USA	Prospective cohort (Nurses’ Health Study)	77,443 women (1992–2010)	Colorectal cancer	Self-reported PD and number of natural teeth	None measured	<17 teeth associated with HR=1.20 for CRC; strongest for proximal colon (HR=1.23) and rectal cancer (HR=1.48)	Tooth loss + PD proxy for long-standing immune imbalance	Moderate
Michaud et al. (2016) [33]	USA	Prospective cohort (HPFS, never smokers)	19,933 men	All cancers (esp. oropharynx, bladder, esophagus)	Self-reported PD + <17 teeth	None measured	Advanced PD with <17 teeth had HR=2.57 for tobacco-related cancers (non-smokers); HR=6.29 for oropharyngeal/esophageal	Even without smoking, PD severity drives systemic oncogenic burden	Moderate

A formal risk of bias assessment was performed for all included studies using NOS and AXIS tools, based on study type. The majority of cohort and case-control studies were rated as low risk, while cross-sectional studies were more frequently judged to carry a moderate risk of bias. Detailed scoring and tool assignment per study are available in Table 2, which complements the main synthesis by contextualizing the robustness of each dataset.

**Table 2:** Risk of bias assessment using validated tools Risk of bias was assessed using validated tools based on study design. The Newcastle-Ottawa Scale (NOS) was used for cohort and case-control studies, while the AXIS tool was applied to cross-sectional studies. Ratings are reported as "low," "moderate," or "high" risk of bias based on criteria defined by each instrument.

Author (year)	Study design	Risk of bias tool	Overall risk of bias
Ahn et al. (2012) [12]	Cohort	NOS	Low
Kajihara et al. (2022) [3]	Cross-sectional	AXIS	Moderate
Kim et al. (2022) [18]	Cohort	NOS	Low
Mai et al. (2016) [19]	Cohort	NOS	Low
Ortiz et al. (2022) [20]	Cross-sectional	AXIS	Moderate
Qi et al. (2020) [21]	Cohort	NOS	Low
Saleh et al. (2025) [22]	Cross-sectional	AXIS	Moderate
Uçan Yarkaç et al. (2024) [23]	Cross-sectional	AXIS	Moderate
Heikkilä et al. (2018) [24]	Cohort	NOS	Low
Gao et al. (2016) [25]	Case-control	NOS	Moderate
Fan et al. (2018) [26]	Nested case–control	NOS	Low
Chang et al. (2016) [27]	Cohort	NOS	Low
Chung et al. (2016) [9]	Cohort	NOS	Low
Castellarin et al. (2012) [28]	Case-control	NOS	Moderate
Freudenheim et al. (2016) [29]	Cohort	NOS	Low
Hu et al. (2018) [30]	Cohort	NOS	Low
Michaud et al. (2018) [31]	Cohort	NOS	Low
Momen-Heravi et al. (2017) [32]	Cohort	NOS	Low
Michaud et al. (2016) [33]	Cohort	NOS	Low

Periodontitis and Types of Cancer

These studies represented a broad range of cancers, with the most frequent associations reported in gastrointestinal, respiratory, and genitourinary malignancies. Lung, colorectal, and pancreatic cancers appeared most often and were typically linked with moderate to severe periodontitis.

Notably, several studies identified associations between periodontal inflammation and specific cancer biomarkers or histological subtypes. For example, one study reported elevated PSA levels in association with periodontitis. In contrast, others highlighted strong correlations with malignancies of microbial relevance, such as oropharyngeal and esophageal cancers. These observations suggest a potential gradient of association, whereby chronic oral inflammation may exert differential effects depending on tumor site, oncogenic pathway, or microbial susceptibility.

Inflammatory and Immune Mechanisms

Several studies have investigated specific immunoinflammatory pathways that may mediate the observed associations. Frequently assessed markers included IL-6, TNF-α, regulatory T-cells (Tregs), and CRP, alongside microbial antigens such as *Porphyromonas gingivalis* and *Fusobacterium nucleatum*.

Elevated systemic cytokine levels and immune cell dysregulation were consistently observed among individuals with both periodontitis and cancer. These findings support the hypothesis that chronic periodontal inflammation may trigger systemic immune activation, thereby creating a microenvironment conducive to tumor initiation and progression.

**Figure 2:**
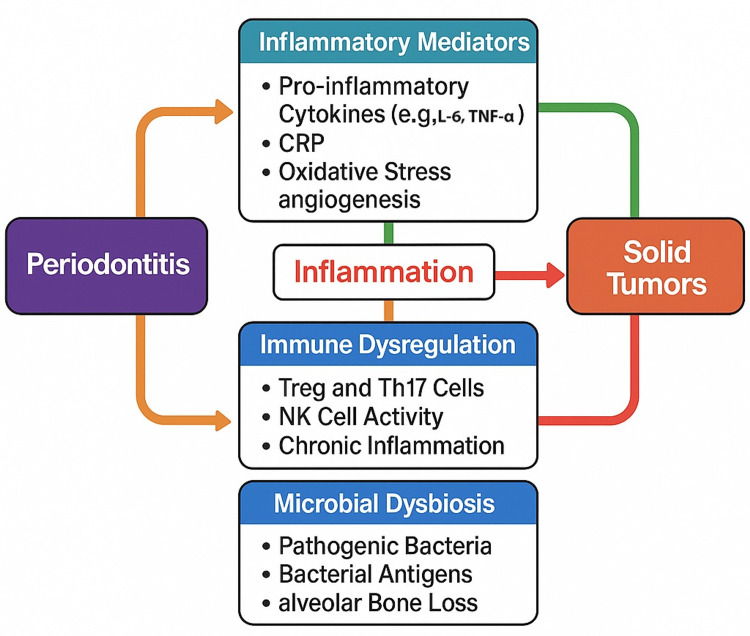
Conceptual framework illustrating immunoinflammatory mechanisms connecting periodontitis with solid tumors The figure highlights key cytokines, immune cell pathways, and microbial agents implicated in the systemic carcinogenic process. Image Credit: Authors

A conceptual synthesis of these mechanisms is provided in Figure 2, which outlines the immunoinflammatory and microbial pathways potentially linking periodontal disease to cancer development.

Evidence Synthesis and Consistency

Across the reviewed studies, a consistent pattern emerged suggesting a positive association between periodontitis and elevated cancer risk. While causality cannot be established due to the observational nature of the data, the convergence of findings from large, well-adjusted cohorts, combined with mechanistic plausibility, lends credibility to the relationship.

Most studies were classified as providing moderate levels of evidence. Limitations included heterogeneity in definitions of exposure and outcomes, the presence of potential confounders, and reliance on surrogate markers. Nevertheless, the overarching narrative supports a biologically plausible and clinically meaningful link between chronic periodontal inflammation and solid tumor pathogenesis.

Discussion

Summary of Main Findings

This systematic review synthesized original clinical research investigating immunoinflammatory mechanisms that may link periodontitis to the development or progression of solid tumors. Nineteen studies met the inclusion criteria. Despite variability in study design, populations, and methodologies, a consistent pattern emerged: periodontitis is associated with elevated systemic inflammation and immune dysregulation, conditions conducive to tumorigenesis [3,4,8,11,13].

Most studies provided moderate-level evidence supporting associations between periodontal disease and malignancies such as colorectal, pancreatic, lung, and prostate cancers. Repeatedly elevated inflammatory markers, including IL-6, TNF-α, and CRP, as well as regulatory T-cells, were observed in individuals with periodontitis, indicating a potentially shared pathogenic environment with certain tumors [3,8,9,13].

Microbial dysbiosis, particularly involving *Porphyromonas gingivalis* and *Fusobacterium nucleatum*, was also identified as a plausible mechanistic pathway. These microbes may exert local effects in the oral cavity and systemic effects following translocation, contributing to immune modulation and inflammatory signaling in distal tissues [5,15,34-36].

Strengths and Limitations of the Evidence

This review focused exclusively on original clinical studies involving human participants, offering a strong translational foundation for mechanistic interpretation. By emphasizing immunoinflammatory pathways, it advances beyond epidemiological correlation to propose biologically grounded explanations [25,37-39].

Nonetheless, limitations must be acknowledged. Diagnostic definitions of periodontitis varied widely, ranging from radiographic assessments to self-reported measures, introducing potential inconsistency. Cancer outcomes were likewise heterogeneous, with several studies using proxy endpoints such as PSA levels or mortality instead of histological confirmation [8,10,24].

Additionally, detailed immune profiling was often absent, and mechanistic inferences were frequently drawn indirectly rather than through direct measurement. As such, the body of evidence is constrained by its observational nature, susceptibility to confounding, and a general lack of longitudinal designs capable of establishing causality [23,39,40].

Furthermore, this review addressed prior methodological concerns by implementing a structured risk of bias assessment using validated instruments (NOS and AXIS). This step facilitates a more accurate assessment of the internal validity of the included studies. Nonetheless, limitations persist, particularly for cross-sectional designs where causal inference is inherently restricted. While no study was rated as high risk, several presented moderate concerns regarding selection bias or incomplete exposure ascertainment. These elements should inform cautious interpretation, especially when generalizing findings to diverse populations or clinical settings.

Comparison With Existing Literature

Previous reviews have predominantly focused on epidemiologic trends or microbial hypotheses. In contrast, this review emphasizes the immunological context, aligning with broader literature on inflammation-driven carcinogenesis. Chronic low-grade inflammation and immune evasion, hallmarks of both periodontitis and tumor progression, may share regulatory pathways, as suggested by findings from the included studies [8,9,13].

Implications for Clinical Practice

From a clinical perspective, these findings support the hypothesis that mitigating periodontal inflammation may offer systemic benefits, potentially including a reduced risk of cancer. Periodontitis is a modifiable risk factor, particularly in populations with immune vulnerabilities or pre-existing pro-inflammatory conditions [8-10]. However, it is premature to infer causality or advocate for periodontal treatment as a preventive oncology measure without further mechanistic validation and longitudinal evidence [8,9,15].

Recommendations for Future Research

Future investigations should standardize diagnostic criteria for periodontitis and integrate biomarker-based immune profiling into study protocols. Longitudinal cohort studies tracking both immunoinflammatory markers and cancer outcomes are needed to strengthen causal inference. Randomized trials assessing the systemic effects of periodontal therapy on inflammatory biomarkers would further enhance understanding of potential interventional benefits [1,23,24].

Conceptual Integration

**Figure 3:**
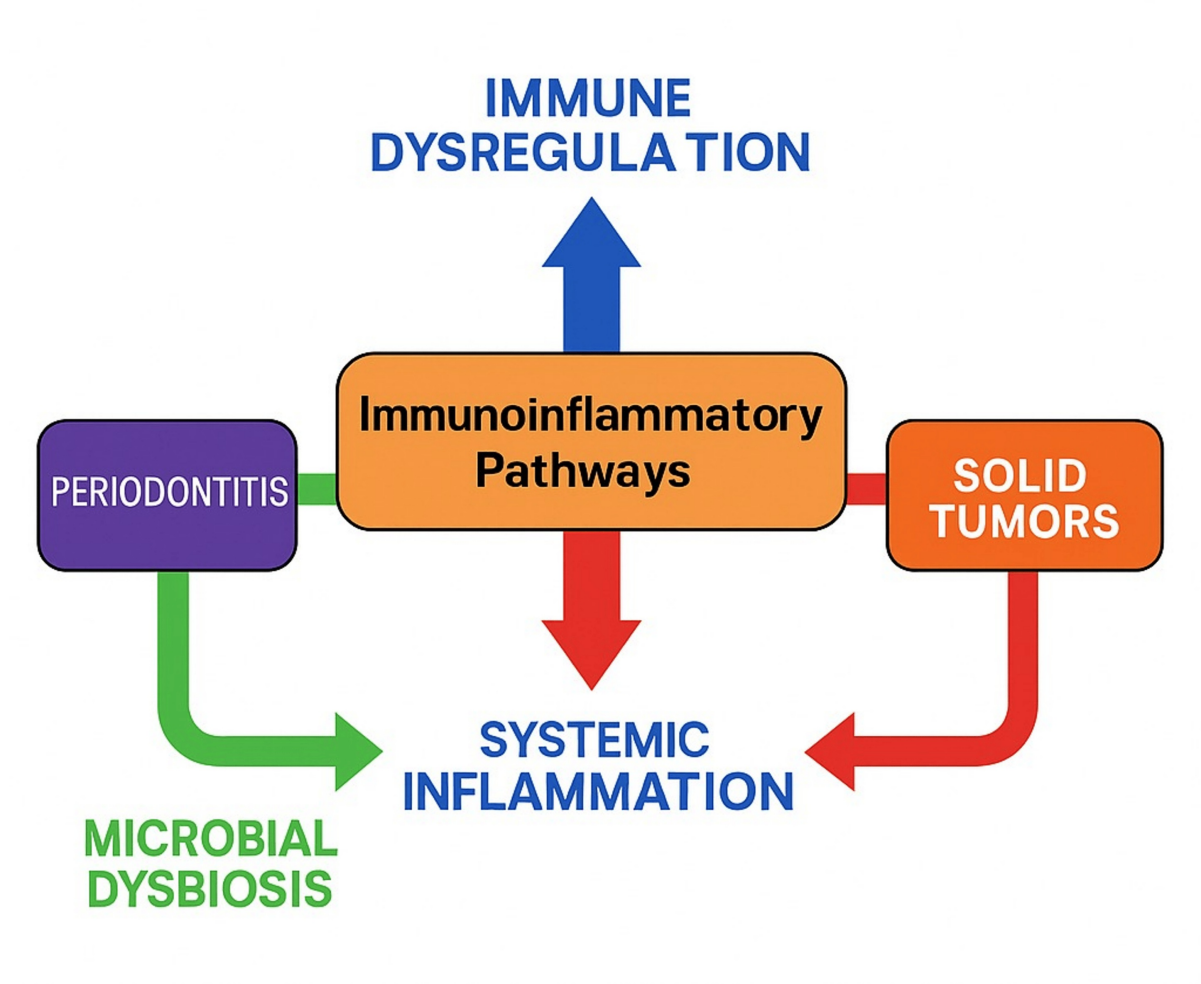
Conceptual framework summarizing immunoinflammatory pathways potentially linking periodontitis and solid tumors It highlights microbial dysbiosis, systemic inflammation, and immune dysregulation as interconnected drivers of tumor-promoting conditions. Image Credit: Author

A synthesized conceptual model is presented in Figure 3, integrating microbial, immune, and inflammatory axes derived from the reviewed studies. This framework illustrates how these interconnected pathways may converge to influence tumor biology.

## Conclusions

This systematic review integrated original clinical research exploring immunoinflammatory pathways that may underlie the association between periodontitis and solid tumors. Among the 19 studies included, a consistent narrative emerged: chronic periodontal inflammation is linked to systemic immune dysregulation, microbial translocation, and elevated levels of pro-inflammatory mediators-conditions that may collectively foster a tumor-permissive microenvironment. Key mechanisms identified include increased circulating cytokines, such as IL-6 and TNF-α, elevated levels of regulatory T-cells, impaired immune surveillance, and the presence of oral pathogens in extraoral sites. These findings support the hypothesis that periodontitis may function as a systemic contributor to inflammation-driven carcinogenesis.

Although the body of evidence is compelling, it remains predominantly observational. Variability in diagnostic criteria for both periodontitis and cancer, as well as differences in study designs and populations, limit the ability to infer causality with certainty. Nevertheless, the convergence of immunological, microbial, and inflammatory evidence highlights a biologically plausible connection that merits continued investigation. From a clinical standpoint, this review highlights the systemic importance of oral health and advocates for a more comprehensive and integrated approach to patient care. Inflammatory processes originating in the oral cavity may have extensive implications, including a potential role in modulating cancer risk. Future research should prioritize longitudinal and interventional studies incorporating standardized periodontal diagnostics and detailed immune profiling. Such approaches are necessary to clarify causal pathways and assess the potential of periodontal interventions to influence systemic disease outcomes. Ultimately, advancing our understanding of the immunoinflammatory interface between periodontitis and cancer could pave the way for innovative preventive and therapeutic strategies that bridge the divide between oral and systemic health.

## References

[1] Hajishengallis G, Chavakis T (2021). Local and systemic mechanisms linking periodontal disease and inflammatory comorbidities. Nat Rev Immunol.

[2] Hirschfeld J, Higham J, Blair F, Richards A, Chapple IL (2019). Systemic disease or periodontal disease? Distinguishing causes of gingival inflammation: a guide for dental practitioners. Part 2: cancer related, infective, and other causes of gingival pathology. Br Dent J.

[3] Kajihara R, Sakai H, Han Y (2022). Presence of periodontitis may synergistically contribute to cancer progression via Treg and IL-6. Sci Rep.

[4] Karmakar S, Kar A, Thakur S, Rao VU (2020). Periodontitis and oral Cancer-A striking link. Oral Oncol.

[5] Hajishengallis G (2015). Periodontitis: from microbial immune subversion to systemic inflammation. Nat Rev Immunol.

[6] Pan W, Wang Q, Chen Q (2019). The cytokine network involved in the host immune response to periodontitis. Int J Oral Sci.

[7] Plemmenos G, Evangeliou E, Polizogopoulos N, Chalazias A, Deligianni M, Piperi C (2021). Central regulatory role of cytokines in periodontitis and targeting options. Curr Med Chem.

[8] Baima G, Minoli M, Michaud DS, Aimetti M, Sanz M, Loos BG, Romandini M (2024). Periodontitis and risk of cancer: mechanistic evidence. Periodontol 2000.

[9] Chung SD, Tsai MC, Huang CC, Kao LT, Chen CH (2016). A population-based study on the associations between chronic periodontitis and the risk of cancer. Int J Clin Oncol.

[10] Corbella S, Veronesi P, Galimberti V, Weinstein R, Del Fabbro M, Francetti L (2018). Is periodontitis a risk indicator for cancer? A meta-analysis. PLoS One.

[11] Hujoel PP, Drangsholt M, Spiekerman C, Weiss NS (2003). An exploration of the periodontitis-cancer association. Ann Epidemiol.

[12] Ahn J, Segers S, Hayes RB (2012). Periodontal disease, Porphyromonas gingivalis serum antibody levels and orodigestive cancer mortality. Carcinogenesis.

[13] Fitzpatrick SG, Katz J (2010). The association between periodontal disease and cancer: a review of the literature. J Dent.

[14] Binder Gallimidi A, Fischman S, Revach B (2015). Periodontal pathogens Porphyromonas gingivalis and Fusobacterium nucleatum promote tumor progression in an oral-specific chemical carcinogenesis model. Oncotarget.

[15] Javed F, Warnakulasuriya S (2016). Is there a relationship between periodontal disease and oral cancer? A systematic review of currently available evidence. Crit Rev Oncol Hematol.

[16] Wells GA, Shea B, O'Connell D (2024). The Newcastle-Ottawa Scale (NOS) for assessing the quality of nonrandomised studies in meta-analyses. https://www.ohri.ca/programs/clinical_epidemiology/oxford.asp.

[17] Downes MJ, Brennan ML, Williams HC, Dean RS (2016). Development of a critical appraisal tool to assess the quality of cross-sectional studies (AXIS). BMJ Open.

[18] Kim EH, Nam S, Park CH (2022). Periodontal disease and cancer risk: a nationwide population-based cohort study. Front Oncol.

[19] Mai X, LaMonte MJ, Hovey KM, Freudenheim JL, Andrews CA, Genco RJ, Wactawski-Wende J (2016). Periodontal disease severity and cancer risk in postmenopausal women: the Buffalo OsteoPerio study. Cancer Causes Control.

[20] Ortiz AP, Acosta-Pagán KT, Oramas-Sepúlveda C (2022). Oral microbiota and periodontitis severity among Hispanic adults. Front Cell Infect Microbiol.

[21] Qi J, Zihang Z, Zhang J, Park YM, Shrestha D, Jianling B, Merchant AT (2020). Periodontal antibodies and all-cause and cardiovascular disease mortality. J Dent Res.

[22] Saleh MH, Kalani K, Sabri H (2025). Association between periodontitis severity and prostate-specific antigen levels using the NHANES data. J Periodontol.

[23] Uçan Yarkaç F, Babayiğit O, Gokturk O (2024). Associations between immune-inflammatory markers, age, and periodontal status: a cross-sectional study. Odontology.

[24] Heikkilä P, But A, Sorsa T, Haukka J (2018). Periodontitis and cancer mortality: register-based cohort study of 68,273 adults in 10-year follow-up. Int J Cancer.

[25] Gao S, Li S, Ma Z (2016). Presence of Porphyromonas gingivalis in esophagus and its association with the clinicopathological characteristics and survival in patients with esophageal cancer. Infect Agent Cancer.

[26] Fan X, Alekseyenko AV, Wu J (2018). Human oral microbiome and prospective risk for pancreatic cancer: a population-based nested case-control study. Gut.

[27] Chang JS, Tsai CR, Chen LT, Shan YS (2016). Investigating the association between periodontal disease and risk of pancreatic cancer. Pancreas.

[28] Castellarin M, Warren RL, Freeman JD (2012). Fusobacterium nucleatum infection is prevalent in human colorectal carcinoma. Genome Res.

[29] Freudenheim JL, Genco RJ, LaMonte MJ (2016). Periodontal disease and breast cancer: prospective cohort study of postmenopausal women. Cancer Epidemiol Biomarkers Prev.

[30] Hu JM, Shen CJ, Chou YC (2018). Risk of colorectal cancer in patients with periodontal disease severity: a nationwide, population-based cohort study. Int J Colorectal Dis.

[31] Michaud DS, Lu J, Peacock-Villada AY (2018). Periodontal disease assessed using clinical dental measurements and cancer risk in the ARIC study. J Natl Cancer Inst.

[32] Momen-Heravi F, Babic A, Tworoger SS (2017). Periodontal disease, tooth loss and colorectal cancer risk: results from the Nurses' Health Study. Int J Cancer.

[33] Michaud DS, Kelsey KT, Papathanasiou E, Genco CA, Giovannucci E (2016). Periodontal disease and risk of all cancers among male never smokers: an updated analysis of the health professionals follow-up study. Ann Oncol.

[34] Bregaint S, Boyer E, Fong SB, Meuric V, Bonnaure-Mallet M, Jolivet-Gougeon A (2022). Porphyromonas gingivalis outside the oral cavity. Odontology.

[35] Geng F, Liu J, Guo Y (2017). Persistent exposure to porphyromonas gingivalis promotes proliferative and invasion capabilities, and tumorigenic properties of human immortalized oral epithelial cells. Front Cell Infect Microbiol.

[36] Yao L, Jermanus C, Barbetta B, Choi C, Verbeke P, Ojcius DM, Yilmaz O (2010). Porphyromonas gingivalis infection sequesters pro-apoptotic Bad through Akt in primary gingival epithelial cells. Mol Oral Microbiol.

[37] Coussens LM, Werb Z (2002). Inflammation and cancer. Nature.

[38] Higgins JP, Thompson SG, Deeks JJ, Altman DG (2003). Measuring inconsistency in meta-analyses. BMJ.

[39] Kapila YL (2021). Oral health's inextricable connection to systemic health: special populations bring to bear multimodal relationships and factors connecting periodontal disease to systemic diseases and conditions. Periodontol 2000.

[40] Taubman MA, Valverde P, Han X, Kawai T (2005). Immune response: the key to bone resorption in periodontal disease. J Periodontol.

